# The nitro-chloro substitution on two quinolinone-chalcones: from molecular modeling to antioxidant potential

**DOI:** 10.1063/4.0000811

**Published:** 2025-10-27

**Authors:** Renata Layse G. de Paula Master, Jean M. F. Custódio, Caridad Noda-Pérez, Allen G. Oliver, Hamilton B. Napolitano

**Affiliations:** 1 Universidade Estadual de Goiás, Anápolis, Goiás, Brazil; 2 University of Notre Dame, Notre Dame, Indiana, USA; 3 Universidade Federal de Goiás, Goiânia, Goiás, Brazil

## Abstract

The current demand for sustainable energy sources leads us to consider the possibility of biofuels, such as biodiesel, which are more environmentally desirable and align with some of the Sustainable Development Goals.^¹^ Biodiesel stands out as a more environmentally friendly option, as it presents lower levels of pollutants during combustion. However, its low oxidative stability represents a significant challenge, as it accelerates its degradation process, reducing its useful life and compromising its performance when compared to petroleum-derived fuels.^²^ In this context, quinolinone-derived compounds, such as quinolinone-chalcones, have shown interest in research into biofuel additives. These bicyclic heterocycles have chemical structures that favor electronic interactions capable of neutralizing reactive species, giving them potential antioxidant activity. This property is highly desirable for biodiesel additives, as it can mitigate premature oxidation of the fuel, prolonging its stability and enabling its use on a large scale.^³^ The title compounds were synthesized via Claisen–Schmidt condensation between 2’N- phenylsulfonylacetophenone and a benzaldehyde, using basic catalysis in an ethanolic medium.^4^ Two crystallographic structures of quinolinone-chalcones (QC-NO2 and QC-Cl; Figure 1a and 1b) have been analyzed. The substitution of the nitro group in QC-NO_2_ results in distinct interactions and, consequently, a different molecular packing (Figure 1c). An overlay of this region with nitro-chloro substitution shows an RMSD of 0.0421, as illustrated in Figure 1d. The chlorine atom led to intermolecular chlorine…hydrogen interactions (8.8%), which directed the supramolecular arrangement. The molecular modeling of nitro-chloro substitution was evaluated by Frontier molecular orbitals (Highest Occupied Molecular Orbital - Lowest Unoccupied Molecular Orbital), Molecular electrostatic potential MEP map, Fukui indices, QTAIM analysis and Hirshfeld surfaces. The analyses indicated that the substitution of the chlorine group (QC-Cl) by nitro in QC-NO_2_ also resulted in a higher number of C–H…O interactions (45.0%) compared to QC-Cl (29.5%), which may directly influence the molecular stability and its ability to donate electrons or hydrogen atoms, neutralizing free radicals and interrupting oxidation chain reactions (antioxidant activity).
